# Adequacy and Distribution Equity of Nutrition Supplies across China

**DOI:** 10.3390/nu16030426

**Published:** 2024-01-31

**Authors:** Chuan Zhao, Zhengyang Zhang, Kazuyo Matsubae

**Affiliations:** 1 Graduate School of Environmental Studies, Tohoku University, Sendai 980-8577, Japan; zhao.chuan.t7@dc.tohoku.ac.jp (C.Z.); kazuyo.matsubae.a2@tohoku.ac.jp (K.M.); 2 Research Institute for Humanity and Nature, Kyoto 603-8047, Japan

**Keywords:** nutrition supply, nutrient flow, diet intake, local supply, trade

## Abstract

Procuring food enriched with diverse nutrients is pivotal for maintaining a robust immune system. However, the food system is now unprecedentedly globalized and faces challenges arising from climate change, pandemics, and political unrest. This study aims to illuminate the gap in exploring the adequacy and distribution equity of nutrition supplies in response to potential trade fluctuations and restrictions on agrifood within China’s local agriculture endowments. Also, it seeks to identify the role of trade in contributing to these indices. Accordingly, we analyzed the distribution of nutrients in agrifood categories from production to consumption and assessed the adequacy and distribution equity of corresponding available nutrition supply from the local food provision system in terms of meeting residents’ nutritional requirements, across China, and compared with those in the practical market. The local self-supply system showed that 12 out of 31 provinces have difficulty achieving an iron supply with 11% to 108% deficiencies. Except for iron, 52% (folate)–90% (vitamin B12) of agricultural output was available for diet provisioning nationwide. While food trade emerges as a crucial factor in enhancing secure and equitable nutrition supply, risks associated with micronutrient deficiencies necessitate careful consideration in current global circumstances. Our analysis explored a regional pool of nutrient information in supplements to the conventional food profile in China and could implicate better knowledge toward healthier food supplies and tailored improvements for achieving a resilient nutrition supply.

## 1. Introduction

Maintaining human health requires a wide range of nutrition from the food supply to support a functional immune system [1]. The shift from calorie-focused food security to nutrition security arises from a better understanding of the link between diet and illnesses, particularly micronutrient deficiencies [2]. There is a growing consensus that enhancing nutrient optimization in the food system can address food deficits more effectively than solely increasing food production [3,4]. 

In addition, the challenges faced by the global food system, including climatic shocks, pandemics, and political unrest, magnify risks in coping with secure and shared nutrition supply [5,6]. The food system undergoes significant changes due to agricultural advancement and increasing urbanization, prompting debates on merits and perils of localized versus globalized food systems, across all angles and geographies to reach a universally applicable conclusion [7]. The transition of food systems worldwide from rural-led to industrialized and intensified systems is characterized by extended production-to-consumption distances and more granular joints such as production, processing, storage and transport, retail, consumption, and waste disposal [8]. Parallel to this, the debate stemming from production–consumption distances, implying an autonomous local food system versus a globalized system reliant on international trade, has never been agreed upon on all fronts. For example, proponents of the global system assert that food trade enhances global food security, while proponents of localization claim that increased local production and consumption can support local economies, social equity, and nutritional health [9,10,11,12]. Urbanization, external transport reliance, and recent global events like the Coronavirus outbreak and conflicts impact food supply chains and emphasize the need to examine the supply capacity and vulnerability of diverse food systems. 

In recent decades, China has undergone a significant transition in agriculture, urbanization, and diet structure, marked by a sharp rise in demand for animal-based foods and an increased risk of health problems due to excessive energy intake and unbalanced diets [13,14]. Moreover, the temporary swings in the domestic food supply at the beginning of the COVID-19 outbreak and the call for innovation in the food system post-pandemic have also prompted the extensive consideration of localization and global coordination in healthier nutrition supply [3,15]. However, information on the local nutrition supply potentials and the role of trade considering the nutritional gap and related equitable distribution is still incomplete at the subnational level in China (more information is provided in the following Section 2). The absence of this information could impede the formulation of more effective policies and strategies aimed at fostering a healthier, more resilient, and equitable nutrition supply across China.

To address these issues, our study strives to (1) conduct a provincial-level evaluation in China, assessing the theoretical local nutrition supply capacity by tracing nutrient flows from farm to fork, following the approach of the nutrient balance sheet as outlined by Lavidini and Masters [16]; (2) evaluate the equities of local nutrition distribution; (3) compare nutrition supply adequacies and distribution equities with those in the practical market to unveil the impact of trade and assess the resilience of local self-supply to shocks. The term “practical market” refers to the practical nutrition supply, encompassing intranational and international trade consequences.

## 2. Literature Review

In this section, we organized a literature review regarding the public concern shift from food security to nutrition security and the existing studies on assessing nutrition supply at different scales, aiming to elucidate the importance of these assessments and address pertinent research gaps.

*From food security to nutrition security.* The discourse on food security, rooted in the philosophy established at the 1996 World Food Summit, underscores the imperative significance of agricultural productivity [17]. Global solidarity in agriculture policy and technology investment has brought farm productivity to about keeping pace with global population expansion, serving 79% of people off hunger compared with 55% five decades ago [18,19]. Still, the greater resolution remains obliged to guarantee the food system to satiate the expected 10 billion people by 2050 under ecological pressures associated with acute land shortages, biodiversity loss, and deteriorating air quality [20,21]. 

In the latest decades, discussion on food security that primarily and chronically focused on calories (aka energy intake) has gradually shifted toward nutrition security as a fact of prevalent micronutrient deficiencies that pose a double predicament of malnutrition that used to be overlooked [22,23]. The dilemma of diet-related nutritional health aggregates risks to the global burden of diseases: more than two billion individuals worldwide lack access to adequate nutrients, while another two billion individuals have been suffering from an overweight status or obesity, making malnutrition a paradoxical “new normal” and growing concern [22,24,25].

*Assessment of nutritional supply.* Elevating the assessment of nutritional supply capabilities is essential for ensuring food security [26]. A full accounting of agricultural production, trade, and distribution from each product, reflected in the national food balance sheets maintained by the Food and Agriculture Organization of the United Nations, facilitates the monitoring and policy guidance of the food system [16]. And they are increasingly adopted to track nutrition supplies through various food systems globally [16,27,28], regionally [29,30,31], and nationally [32,33,34], emphasizing the significance of nutrition accounting in monitoring and intervening in food systems, and highlighting the need to consider nutritional connections from agricultural production through trade and processing before consumption. 

For instance, by assessing the micronutrient intensity of the food supply and the occurrence of inadequate intake of micronutrients, Beal et al. [27] portrayed the global dietary quality trend and discovered that calcium, iron, vitamin A, and zinc are in the lowest levels of adequate micronutrient uptake with significant regional and national variations. Lividini and Masters presented a comprehensive nutrient balance sheet encompassing all flows of plant- and animal-based foods, spanning from farm production and trade to non-food applications and waste, across 173 countries [16]. The analysis offered substantial changes required to ensure sufficient supplies of essential nutrients by adjusting agricultural production toward nutrient-dense commodities, which can be achieved through strategies like the biofortification of locally grown crops, fortification of packaged foods available in local markets, or targeted supplement delivery to specific subpopulations. Strides are also made regarding the impacts of market shocks or trade restrictions on nutrition supply. 

In an attempt to determine the national nutrition gaps between supply and demand, Liu and colleagues demonstrated that the nutrition supplies in China’s food system had increased sharply over the past five decades, and some elements like calcium and zinc need further improved provisioning [33]. In addition, utilizing the food-to-nutrient transformation model, the researchers computed nutrient production and consumption, along with nutritional adequacy pertaining to energy, protein, and fat, across 11 coastal provinces in China [35]. An examination of spatial patterns led to the conclusion that the studied region exhibited satisfactory protein levels but faced deficiencies in both energy and fat levels. However, studies are constrained either at differentiating spatial variation or limited nutrients. Moreover, Wood et al. [36] created a postulated non-trade scenario to approximate and compare the effect of the current international trading system on nutritional availability. They argued that without trade, many countries—particularly low-income countries—would be less able to meet their nutritional needs and suggested that transaction is associated with better equitable access to nutrition in the current global food system. Nevertheless, limited studies have been conducted on local agrifood systems for informing a range of interventions in response to the vulnerability of diverse food systems to trade fluctuations at a subnational scale. This analysis aims to supplement fundamental information on the local agrifood system in China and provide quantitative data to support potential policy scenarios for stakeholders and policymakers in responding to market shocks.

## 3. Methods and Materials

### 3.1. Data Source

We correlated the provincial food flows, nutrient constituents to food categories, and nutrient requirements of specific age groups to assess China’s food-nutrient supply adequacy and distribution equitability. Using pairwise scenarios, we compared the effects of a practical market on local nutrient supply regarding food system resilience and equitable access to nutrients (Figure S1). The detailed approaches are presented in Section 3.2, Section 3.3 and Section 3.4.

*Food classification and nutrients of interest.* In this study, agrifood production profiles were extracted and compiled from statistical yearbooks by the National Bureau of Statistics of China, which comprehensively and representatively report the Chinese agricultural inputs and outputs. We traced functional flows of each food type, including crops and animal-based products, from production to supply, i.e., total productivity, conversion to seed and feed, food losses, and non-food use, alongside available food supply for consumption. Food categories were then aggregated and classified into nine groups: grains, root and tubers, beans, vegetables, fruits, meats, eggs, milk, and seafood, while beverages, peppers, and some others were excluded. Here, we focus on protein, calcium, iron, zinc, folate, vitamin B12, vitamin A, vitamin C, and energy, representing the calorie intake, and covering the least secure and most considerable for both macro and micronutrient intake in China.

*Data* on provincial agricultural profiles related to productivity and cultivated area that are used to estimate food flows and household food consumption were sourced from the National Bureau of Statistics in China, *China Agricultural Yearbook*, and *China Food Industry Yearbook* [37,38,39]; population information was extracted from the seventh national census [40]. Food items’ nutrient composition and refuse values are according to the nutrient data from USDA Food Composition Database; the database contains up to 400,000 food entries and five major types of information on macronutrients, vitamins, phytonutrients, minerals, and other components [41]. The full data source is listed in Table S1, and the reference year in the current study is 2019. In addition, benchmarks such as the seed and feed conversion, food loss and waste, and non-food use ratio are based on existing studies or FAOSTAT, as mentioned in this section.

### 3.2. Nutrition Requirement

Each age group’s physical needs and behavioral characteristics indicate that the nutritional elements’ conditions differ. The main objectives in this subsection involve weighted averages of individual nutrition requirements based on the nutrient composition, age distribution of the population in each province (or autonomous region or municipality, henceforth referred to as province, shown in Figure S2), and dietary recommendation for each age class (see Table S2 for age classification and corresponding dietary guidance) (Equation (1)). The total population is then linked to calculate provincial nutrient requirements (Equation (2)).

(1) Ni=∑j,  k,6, 9  (Pj×DRj, k×NCk, i)TP

(2) $${PN}_{i}=N_{i}\times TP$$

where $N_{i}$ and ${PN}_{i}$ denote weighted averages of the individual’s requirement for and total population’s demand for nutrient *i* in a province, respectively. $P_{j}$ denotes age group *j* (n = 6: 0–4, 5–9, 10–14, 15–19, 20–64, 65+), and ${DR}_{j,\;k}$ denotes dietary recommendation of food *k* (n = 9: grains, root and tubers, beans, vegetables, fruits, meats, eggs, milk, and seafood) for the age group *j*. ${NC}_{k,\;i}$ is the content of nutrient *i* in food *k*. *TP* is the provincial total population.

### 3.3. Food Nutrient Productivity and Balance Flows

To estimate the total local production capacity of each nutrient element at the provincial scale, we mapped the annual production data of agrifood products in each province with the food nutrient composition database and aggregated each nutrient contained in the various agriproducts. Similarly, the corresponding nutrient balance flows were analyzed by constructing the food flows from farm to fork by functionally following the nutrient balance sheet approach [16], i.e., conversions to seed and feed, non-food use, food loss, and available food supply for the population.

*Seed.* Seed consumption is one of the more stable changes in grain consumption, and its demand is influenced by the area sown [42]. The seed demand is essentially related to the sowing needed for the following year’s grain harvest. Here, we multiply the seed sowing demand per unit area of different grain crops (Table S3) by the cultivated area to access the seed conversion for each province.

*Feed*. The rapid growth in the scale of livestock production and consumption in China has fueled the use of grain for animal feed, which has become a significant component of Chinese grain consumption [3]. To estimate the feed demand, this study examines six livestock products—pork, beef, mutton, poultry, eggs, and fish products from aquaculture. Province-specific feed demand for each crop was estimated by using the total feed served for animal-based products in the province [43] and allocating the sorts of feed requirements for each crop obtained from Chapagain and Hoekstra [44] (Tables S4 and S5). 

*Food loss* refers to the mass of all crop and livestock food commodities, up to and excluding the retail level, that is directly or indirectly discarded, incinerated, or otherwise wholly left from the post-harvest/meat production/supply chain and not re-used for other uses (e.g., animal feed, industrial uses) [45]. We adopted the national food loss ratios for crops and animal-based products incurred during storage, transport, and processing, assuming that the close infrastructure and technology issues lead to loss across the country [46,47]. Apart from food loss, we also consider the refuse part (e.g., non-edible bones and kernels) in the agriproduct to unify the nutrient content with primary and processed products [48]. The analysis does not include the stock variation as it is collectively stored and not for regular provisioning [42].

*Non-food use* refers to the number of commodities used for other industrial purposes, which in this study was calculated based on a respectively fixed proportion of non-food usage of the specific item to its total production from FAOSTAT [49].

*Local nutrient supplies.* According to Wood et al. [36], local nutrient supplies are defined as the biophysical nutrient endowments owing to the local food system in a hypothesis of no import and export, which can be calculated from Equation (1):

(3) $${LNS}_{m,t}=F\times N$$

where $F=f\left(i\times j\right),\;N=n(j^{T}\times t)$, i denotes the row vector of each province, j denotes the column vector of the available supply of each food that equals the balance after shaving seed and feed, food loss, and other use from total production with the edible part in accounting, j^T^ is the transpose of j, t is the column vector of the selected nutrition mass in a specific food.

*Nutrition supplies in the practical market*. Compared with local nutrient supply capacity, we also considered nutrient supply capacities and distribution equity, including inter-provincial and international trade plus. Obtaining data on inter-provincial agricultural trade is less feasible than country-to-country data in international trade. Here, we start with the final consumption structure and outcomes of the available food supply in the practical market scenario and regard overall household consumption, eating out consumption, and food waste as the provincial food supply.

### 3.4. Adequacy and Distribution Equity of Nutrient Supplies

*Adequacy*. To examine the potential that biophysical local nutrient supplies can satiate the population requirements, we adopted the definition of capacity studies summarized as comparing food consumed or demand by one or more regions within a specified geographic area (such as a state or bioregion) to the level of food that is theoretically produced or produced on peri-urban or nearby rural agricultural landscapes [50]. Accordingly, nutrient adequacy measures and tests local food sufficiency potential in terms of nutrition composition, which may have resilient consequences if communities have to rely more heavily on regional resources to satisfy the needs, which can be expressed as the ratio of specific local nutrient supply to the regional population requirement as

(4) $${Ratio}_{nutrient\;adequacy}=available\;nutrient\;supply/nutrient\;requirement\;\;$$

*Distribution equity*. The equity of both macro and micronutrient supply endowment or distribution among provinces was measured using the Gini coefficient, introduced by the Italian economist Gini in the early 20th century as a composite indicator of income disparity among the population [51]. The Gini coefficient equates to zero, where the distribution is absolutely equal, or to one, where the distribution is absolutely uneven [52]. Here, the income concept was altered with nutrition supply as its similar adoption for carbon footprint or energy consumption cases [52,53]:

(5) $$G_{n}=1-\sum_{i=1}^{31}p_{i}(2Q_{i}-w_{i})$$

where *G_n_* is the Gini coefficient of nutrition n among 31 provinces; *i* = 1, 2, …, 31, and the sequence is ordered from the least to most average n supply in a specific province based on the population; *p_i_* and *w_i_* are the proportions of the population and n supply of each province; *Q_i_* is the cumulative proportion of each province’s n supply.

## 4. Results

### 4.1. Nutrient Requirement

**Figure 1:**
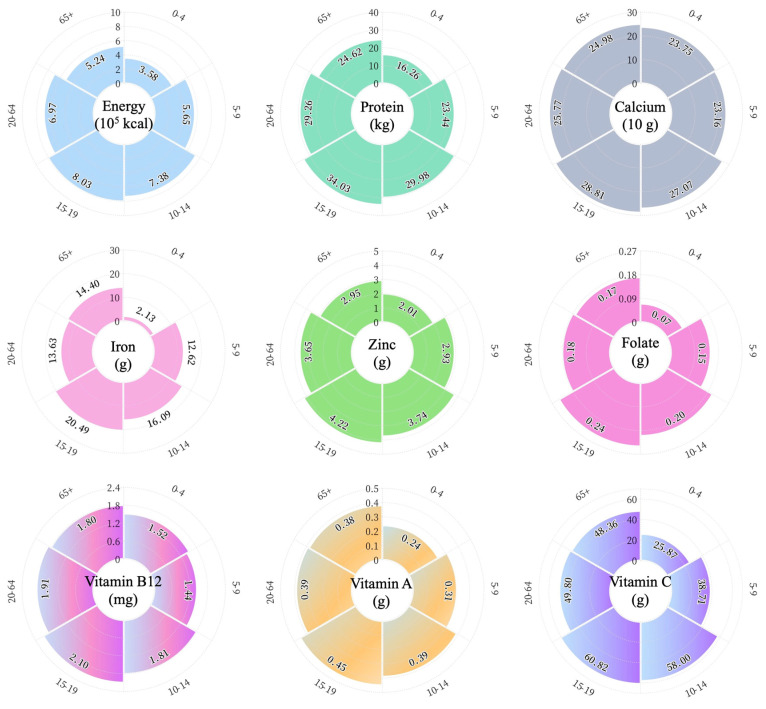
Annual nutrient requirement per capita translated from dietary recommendation for different age groups. The numbers in the outermost ring represent age groups, and the inner numbers indicate the nutritional values.

The annual requirements for the target nutrients for individuals in each age group are presented in Figure 1. In contrast to calcium, concentrated in a narrow range (238–288 g per capita), annual requirements for other nutrients show more significant variability. The yearly requirements for all target nutrients were greatest for 15–19-year-olds, with energy and folate ranging from 9 to 124% and 20 to 243% higher than the rest of the population. Aside from calcium and vitamin B12, infant children aged 0–4 years require the least amount of other nutrients. In addition, we can find that the overall requirements for each nutrient for children under nine years of age and people over 65 years of age are relatively minor compared to for adolescents undergoing rapid growth and for the labor-intensive adult population.

**Figure 2:**
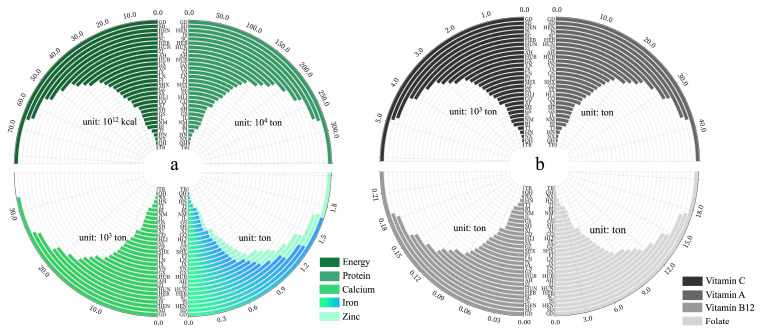
Total nutrient requirements annually in each province. The abbreviation and geographic location can be found in Figure S1. (**a**) Requirements for energy, protein, calcium, iron, and zinc; (**b**) Requirements for vitamin C, vitamin A, vitamin B12, and folate.

Of overall nutrient requirements in each province, the nutrient requirements at the provincial scale, in general, are determined using the total population size (Figure 2). For example, the top five provinces with the largest population (GD, SD, HEN, JS, SC) and the bottom five provinces (TB, QH, NX, HN, TJ) have nutrient requirements ranked in line with their population size (Figure S2). At the same time, there are exceptions due to population structure. For example, GS, with 25.02 million people, has a greater need for energy and calcium than SH, with a smaller population (24.87 million people), while SH, with a larger young adult workforce, has a greater need for protein and vitamins than GS, with a relatively younger population structure.

### 4.2. Total Nutrient Productivity and Local Nutrient Flow

**Figure 3:**
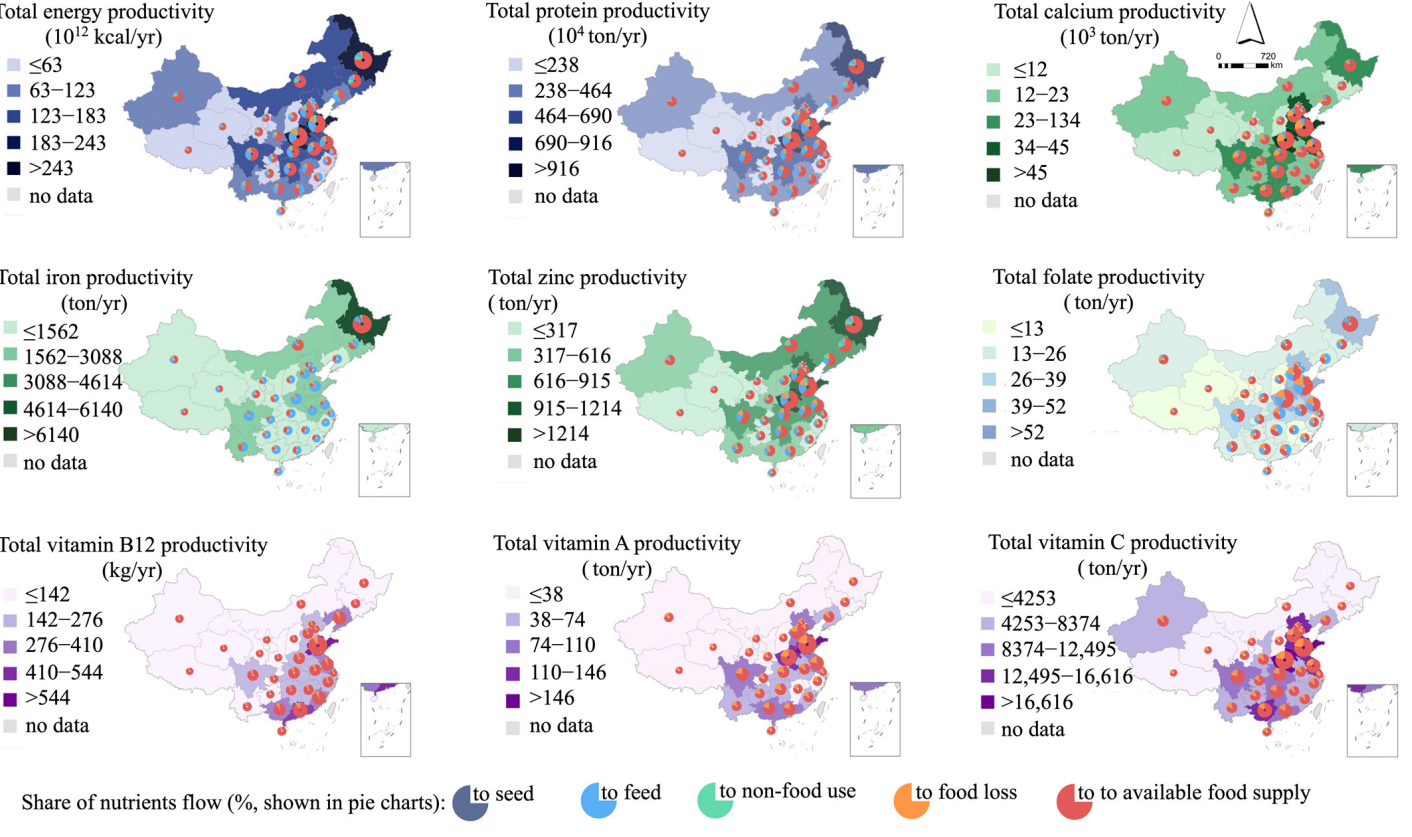
Total nutrient productivity and local nutrient balance flow in China.

On the national scale, the total annual production of energy, protein, calcium, iron, zinc, folic acid, vitamin B12, vitamin A, and vitamin C was 3.1 × 10^15^ kcal, 1.1 × 10^8^ tons, 6.0 × 10^5^ tons, 3.8 × 10^4^ tons, 1.6 × 10^4^ tons, 6.3 × 10^2^ tons, 5.2 tons, 1.5 × 10^3^ tons, and 2.0 × 10^5^ tons, and exhibited high geographical variability at the provincial scale (expressed here as a coefficient of variation, i.e., the quotient of the standard error of the yield and the mean for each province) from 75% (calcium) to 89% (vitamin B12) (Figure 3).

The local nutrient flow is based on the scenario that excludes domestic and foreign trade and relies exclusively on local food production. In the local self-supply system, conversions for seed demands account for the least share of all nutrient flows (less than 2%). The main nutrient flows for non-food uses are concentrated on macronutrients and minerals in the north–east (HLJ, JL, LN) and some northern provinces (NM, SX), ranging from 10–14% for energy, 8–12% for protein, and 12–14% for zinc; the national average losses for energy, protein, mineral elements, and vitamins are around 10%, 10–18%, and 8–21%, respectively. In some provinces, vitamin loss accounts for up to 25% of total production (e.g., folate and vitamin A in SH, vitamin A in ZJ and GS, and vitamin C in JL). Relatively higher loss rates of approximately 20% for vitamins A and C are primarily due to high vegetable and post-harvest fruit loss rates. Feed relies considerably on energy, protein, calcium, zinc, and folate, accounting for 24%, 21%, 6%, 20%, and 28% of their overall production. In addition, in a self-sufficient context, 12 out of 31 provinces have difficulty achieving an iron feed supply with deficiencies of 11% (GX) to 108% (GD). The results (except for iron) show that after removing nutrient losses from seed and feed conversion, non-food usage requirements, and food losses, between 52% (folate) and 90% (vitamin B12) of agricultural output is available for supply on the national level, where the status of the available supply of calcium and vitamins (excluding folate) is broadly similar among the provinces. 

### 4.3. Nutrient Supply in the Practical Market

Combining the final consumption components of nutrient supply, we estimated the supply by provinces in China under the practical market (Table S6). For all the selected nutrients, the majority of supplies were over 80% household-consumed among provinces. For away-from-home consumption, vitamin B12 (8–26%) and A (7–30%) are marginally greater than the other nutrients in the entire consumption composition. The waste rate was approximately 10% of every nutrient supply, except for iron, which was 18% (GS) to 33% (BJ). Also, some location variation was observed for the waste rate of specific nutrients. For example, energy and protein waste rates are relatively higher in the eastern coastal and economically developed parts (e.g., BJ, SH, ZJ, JS, FJ).

### 4.4. Nutrient Supply Adequacy and Distribution Equity among Provinces

**Figure 4:**
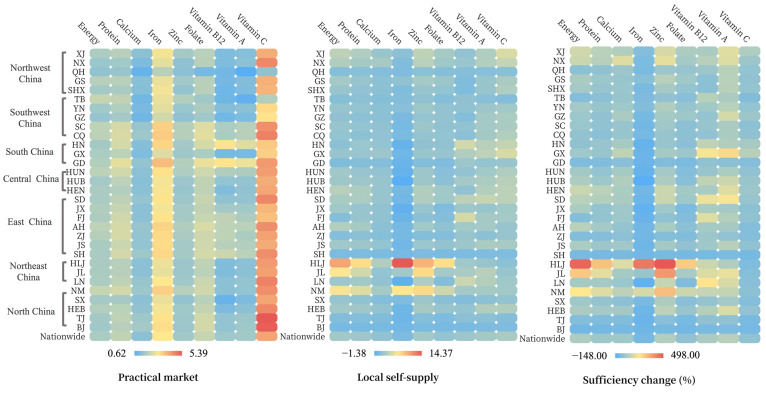
Nutrient adequacies in local self-supply and practical market scenario.

Most places can satisfy their nutrient demands except for iron in the local self-supply system. Specifically, 16% (for vitamins A and C)—42% (for folate) of the total area cannot afford self-sufficiency, depending on the nutrient type. Parts of the provinces have superior supply adequacy of nutrients due to their favorable soil climate. For example, energy, protein, calcium, iron, and zinc rates of supply adequacy are multiple times greater in NM, HLJ, and JL than in other regions. Southwest, South, and Central China have relatively adequate vitamin supply rates. Promisingly, upscaled regional coordination can broadly release these deficiencies (Figure 4). However, 27 of the 31 provinces are not self-sufficient in iron supply, and some areas are severely deficient. 

**Figure 5:**
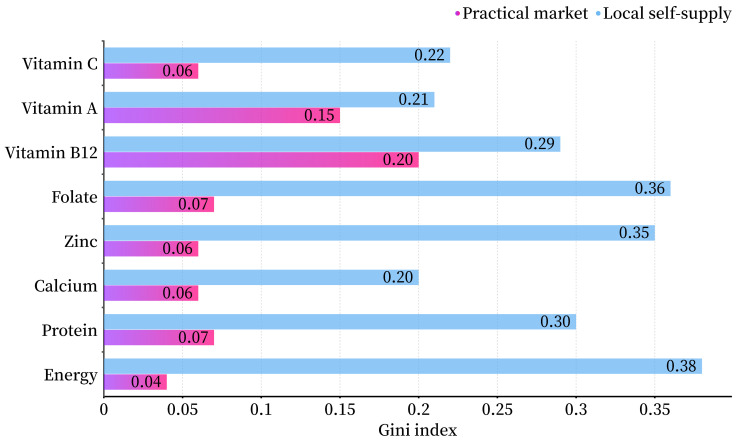
Equitabilities of nutrient distribution in local self-supply and practical market scenario.

Shifted from the practical market to the local scenario, iron supply would drop substantially by 91% while the adequacies of vitamin B12 and A increased slightly by 10%, or 32% on a national average. In addition, trade contributes to reduced inequities in distribution, especially for energy, protein, calcium, zinc, and vitamin C, as reflected in the decline from 0.20 (calcium)—0.38 (energy) to 0.04 (energy)—0.20 (vitamin B12) of the Gini coefficient (Figure 5).

## 5. Discussion

### 5.1. Nutrition Supply and Deficiency in China

Diet has undergone significant changes in China, triggered by economic growth, shifting dietary habits, and increasing globalization. The present study analyses nutritional supply for the diet in different trade scenarios across China. In 2019, for example, in the presence of domestic and international trade, most of the nutrition supply was adequate, and the distribution was relatively equitable; only the supply rate of calcium was slightly less than the required one (>1.5 was considered a valid adequacy rate, assuming a waste rate of 33% at the final consumption stage). The adequacies of national nutrient supplies (e.g., energy, protein, calcium) in the practical market in our results are consistent with Liu et al. [33], who conducted a country-scale analysis for the year 2018 based on FAOSTAT. However, the adequacies of vitamin C (4.7) and vitamin A (3.6) reported in their analysis are much higher than our estimates (4.1 and 1.7 for vitamin C and vitamin A), possibly resulting from accounting approaches, as our estimates of the national average were based on provincial outcomes that consider vegetable and fruit loss during the interprovincial trade. In addition, our results regarding local energy and protein supply adequacies fall in the ranges of that in the study focusing on 11 coastal provinces in China (Table S7) [35]. 

Despite a slight increase in the consumption of calcium-rich dairy products in China over the past few years, the gap between developed countries in Europe and the United States is still considerable due to economic levels and dietary habits [54,55]. The low level of consumption has led to a lack of attention to the supply side and the potential pitfalls. There are several challenges to addressing calcium deficiency in China. These include limited access to calcium-rich foods in some regions, low awareness of the importance of calcium in the diet, and the need for sustained investment in nutrition programs and initiatives [55]. Therefore, concurrently strengthening the supply of calcium and popularizing the intake of calcium-containing foods will be a prerequisite for calcium nutrient safety.

Our analysis further illuminated the regional disparities in practical nutrition supplies. Regional disparities in nutrition supply existed across China over geographic locations, economic development, and cultural differences. Generally, provinces in the more developed coastal regions tend to have better nutrition supplies than those in the less developed interior areas. For example, the coastal province of Guangdong has a higher per capita intake of protein, fat, and vitamins than the interior province of Yunnan. Dietary patterns also contribute to the variation. For example, the northern province of Heilongjiang has a diet of richer protein and fat than the southern province of Guangdong, which has a higher carbohydrate diet. 

Iron deficiency was a common nutritional problem in China [56]. While the adequacy of iron supply with trade participation, from this analysis, is around three compared to the extreme lack of a local cache, the high waste rate at the food consumption stage compared to other elements will be one of the potential risks of insufficient iron intake by the population. Diseases caused by iron deficiency are widespread worldwide, especially among women and children, and also in China [57]. Iron deficiency and anemia prevalence in the country was estimated at around 20%, with higher rates observed in certain provinces and populations [58]. It is believed that the reasons for this situation in the presence of adequate supply rates are not only the inter-individual income and consumption gap but also the low bioavailability due to the co-ingestion of some substances, such as phytates from legumes and nuts, calcium from milk, tannins from tea, and medication [59,60]. Therefore, the national nutrition plan to supply security and nutrition education for the improved resident awareness tends to play an increasing role in the coordination of iron-related health. 

### 5.2. Resilience of Local Nutrition Supply and Role of Trade

Resilience and sustainability have a mutually reinforcing relationship, with sustainability mainly targeting the maintenance of development in the long term, while resilience refers more to the adaptation process to external disturbances [8]. Improving urban food systems’ resilience, recovery, and adaptability in the face of uncertainties, i.e., achieving a resilient transformation of urban food systems, is a significant challenge. In this study, we traced a series of nutrition elements in the agrifood categories through their distribution from production to consumption in the local self-supply system, striving to examine the resilience of nutrition supplies. A clearer nutrient flow (e.g., tracking nutrient loss in food transformation) relative to the food profile was observed, which can contribute to improved nutrition management toward food security. Unexpectedly, some vitamins’ adequacy increases slightly, like vitamins B12 and A. We consider it attributed to the trade surplus caused by the export of a large number of vegetables, as well as the loss of fruits in domestic trade [46,49]. For example, fruit has more than 65% of total losses in storage and transportation compared to cereals with low loss rates [61]. 

Nonetheless, the supply rate of iron, the most severe nutrient in terms of vulnerability, was apt to drop substantially when shifted to the local reliance; this may largely be owing to a tremendous amount of protein and energy from meat production that people need at the considerable cost of iron substitution such as beans for feed [62]. Moreover, monoculture farming requires some places to use a greater proportion of nutrients for other purposes, thereby reducing the availability of nutrients and making it highly vulnerable to supply shocks. Thus, enhanced regional cooperation, long-term effective communication mechanisms, and the sound steering of the population’s diet toward a more balanced structure, especially to avoid excessive meat demand, are instruments to boost the resilience of the local nutritional supply effectively.

Agrifood-related trade contributed to the improved adequacy of nutrition supply and reduced inequity of supply distribution. The reduced Gini coefficient for the distribution of nutrient supply in each province under the practical market does not characterize differences between individuals in the same way as the income equity index; it reflects homogeneity on a spatial dimension associated with the population. Local supply of nutrients may lead to deficiencies or excesses of certain nutrients, as certain areas may not be able to grow or produce certain specific foods. In contrast, in a practical market, people can opt for foods from a wide range of global locations. This makes it more accessible to a wide range of nutrient types, making it easier to opt for a balanced diet and a healthy lifestyle. 

However, practical markets can also lead to food safety issues that need to be regulated and managed by governments and other regulatory entities. Despite the growth of China’s food trade, concerns about food safety have emerged as a major issue that potentially hampers the nutrition supply. Several high-profile food safety scandals occurred, including incidents involving melamine-contaminated milk and tainted meat products; these incidents have triggered increased scrutiny of food production and stricter regulations on food imports [63,64]. Moreover, the COVID-19 pandemic has significantly impacted China’s food trade and global food supply chains [15,65]. At the beginning of the pandemic, China experienced disruptions in food production and transportation, leading to shortages and price increases [15]. The pandemic has also highlighted the imperatives of nutrition security and the need for more resilient food systems.

The resilience strategy against undernutrition and economic downturns is promising to rely on agriculture and food from domestic and innumerable international sources [5,66]. Global shocks like the COVID-19 pandemic and the previous financial crisis necessitate the further consideration of local resilience and vulnerability against market shock out of international cooperation and coordination. For example, fortification and supplementation programs have been a prevalent attempt to sustain nutrition enhancement due to a shortage of local endowments and a lack of sufficient outsourcing from the food trade [16,32].

## 6. Conclusions

In this study, we examined the distribution of various nutrition elements within agrifood categories, tracing their journey from production to consumption in the local self-provisioning system. By juxtaposing this with adequacy and distribution equity in a practical market, our findings underscore the substantial contribution of agrifood trade to increased adequacy, particularly evident in nutrients such as iron, folate, and vitamin C. While local provisioning demonstrated self-sufficiency regarding certain nutrients like energy and protein, this study reveals iron’s vulnerability on both national and provincial scales. This vulnerability arises not only from production pressures driven by the growing demand for meat but also from a high waste rate during the consumption stage.

Notably, although trade enhances nutrient distribution equity, vigilance is required to address vulnerabilities stemming from sudden shocks or shifts in regional and international trade patterns. Concurrently, local nutrition-strengthening initiatives need to be prioritized. Our analysis not only provides subnational nutrient supply information but also contributes to a more comprehensive understanding of the factors influencing resilient nutrition supply.

Moving forward, policymakers and stakeholders should consider targeted improvements, emphasizing optimized localization and the diversification of markets, and promoting changes in dietary habits. By addressing these key aspects, we can enhance the stability and resilience of nutrition supplies, laying the foundation for informed policy decisions and strategic interventions in the future.

*Limitation and future work.* The current study is subject to several limitations, including the exclusion of non-exhausted nutrients and the omission of stock variation, which may result in an underestimation of supply capacity. Furthermore, the local food production modeled in this study assumes a consistent scale and structure of agriculture, whereas in reality, long-term trade impacts could influence these factors. Nonetheless, this work balances the drawback of the simplified model whilst still deriving reasonable information for the vulnerability of local nutrient supply to a short-term market shock. 

Encouragingly, potential research avenues lie in scenario assessments focused on customized adjustments in cropping and livestock production, aimed at optimizing and fortifying local nutrition supplies for increased resilience. Additionally, conducting temporal analyses of provincial nutrition supply holds promise in elucidating trends and shifts in environmental burdens associated with the transfer of nutrition supply through trade. These endeavors contribute to the implications of fostering a robust nutrition supply, beneficial for both human well-being and environmental sustainability.

## Data Availability

Data are contained within the article.

## Supplementary Materials

The following supporting information can be downloaded at: https://www.mdpi.com/article/10.3390/nu16030426/s1. References [67,68] are cited in the Supplementary Materials.
