# Supplementary material for: Adequacy and Distribution Equity of Nutrition Supplies across China
Source: Nutrients. 2024 Jan 31;16(3):426. doi: 10.3390/nu16030426 (PMC10857370; doi:10.3390/nu16030426)
Supplement: Supplementary file 1 [file nutrients-16-00426-s001.zip › nutrients-2813065-supplementary.pdf]

Supporting Information

Adequacy and Distribution Equity of Nutrition Supplies across China

Chuan Zhao 1, Zhengyang Zhang 1,2*, Kazuyo Matsubae 1,2
1 Graduate School of Environmental Studies, Tohoku University, Sendai, 980-8577, Japan
2 Research Institute for Humanity and Nature, Kyoto, 603-8047, Japan
*Corresponding author's email: zhengyang.zhang.a8@tohoku.ac.jp

Table **S1** List of data sources in the study.

Item	Ref.*
Agri-profile (crop production, livestock raised and cultivated area	NBSC, 2020; CAY, 2020; CFIY, 2020
Population and household food consumption	NBSC, 2021; NBSC, 2020
Nutrition composition and edible proportion	USDA Database
Seed conversion rate	Yang et al., 2009
Feed conversion rate	Chapagain and Hoekstra, 2003; Xin et al., 2018
Food loss	Lu et al., 2022; Wang et al., 2023
Non-food use rate	FAOSTAT
Eating-out consumption	Wang et al., 2020; Zeng and Zeng, 2018
Food waste rate	Niu et al., 2022

Table **S2** Dietary intake recommendation for age groups (http://dg.en.cnsoc.org/).

Age	Ref.
0-4	Dietary Guidelines for Chinese Women and Children (2016 version): children 1-3; children 3-6
5-9	Chinese Student Meal Nutrition Standard (WS/T 554-2017): student 6-8
10-14	Chinese Student Meal Nutrition Standard (WS/T 554-2017): student 9-14
15-19	Chinese Student Meal Nutrition Standard (WS/T 554-2017): students 15-17
20-64	Chinese Dietary Guidelines (2016 version)
65+	Dietary Guidelines for the Elderly (WST 556-2017)

Note: Except for the standard version of the Dietary Guidelines for adults, which specifies the recommended quantity of tubers, cereals, and tubers are merged into one group in other recommended dietary levels for the rest of the population. For ease of differentiation, these recommended amounts of grains and tubers are split according to the national target of 30% of staple foods intake devoted by tubers. A similar allocation is implemented in analyzing milk and eggs consumption away from home.

Table **S3** Seed conversion rates in China (Yang, 2009)

	Rice	Maize	Wheat	Soybean	Others
Conversion rate (kg/ha)	75	75	150	75	225

Table **S4** Feeding grain conversion rates of livestock products in China (Xin et al., 2018 and Yang, 2009)

	Pork	Beef	Mutton	Poultry	Eggs	Fish
Conversion rate (kg/kg)	2.7	2.1	2.2	2.3	1.9	1

Table **S5** Composition of animal feeds adopted for the study (Chapagain and Hoekstra 2003).

unit: ton/head	Feed compositions								
Animals	Wheat and products	Barley and products	Maize and products	Oats	Cereals, Other	Peas	Soybeans	Rape and Mustardseed	Other feeds
				Industrial system					
Cattle	0.054	0.533	0.792	0.069	0.034	0.005	0.151	0.058	4.020
Sheep	0.002	0.032	0.004	0.005	0.001	0.001	0.002	0.001	0.703
Goats		0.013	0.006	0.004	0.001		0.002	0.001	0.163
Pigs	0.069	0.39	0.220	0.039	0.004	0.018	0.053	0.048	0.133
Poultry	0.011		0.010				0.003	0.002	0.008
				Grazing system*					
Cattle	0.012	0.129	0.162	0.025	0.007	0.002	0.032	0.013	5.393
Sheep	0.001	0.013	0.003	0.003	0.001	0.001	0.001	0.001	0.416
Goats		0.007	0.003	0.002	0.001		0.001	0.001	0.254
Pigs		0.047			0.140		0.024		0.379
Poultry	0.011		0.010				0.003	0.002	0.008

*Grazing systems are applied in Inner Mongolia, Tibet, Gansu, Qinghai, Ningxia, and Xinjiang. Rest of the provinces is assumed for industrial systems.

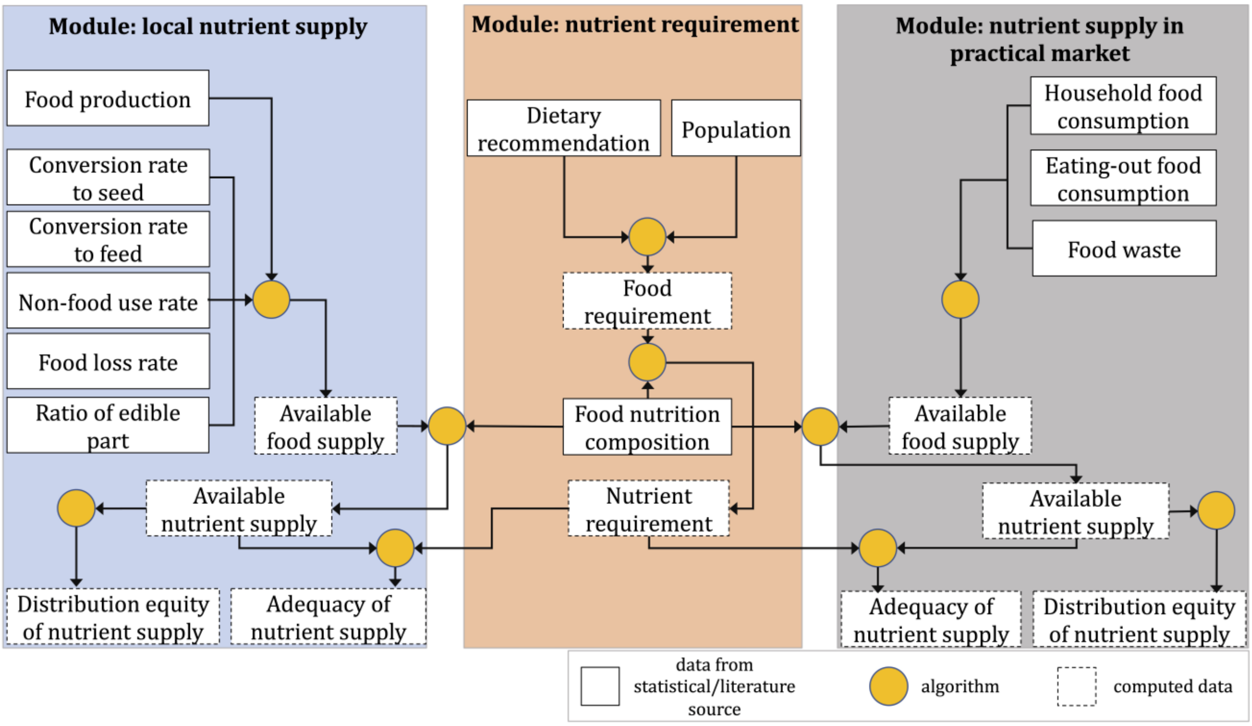

Figure **S1.** Flows of data in this study.

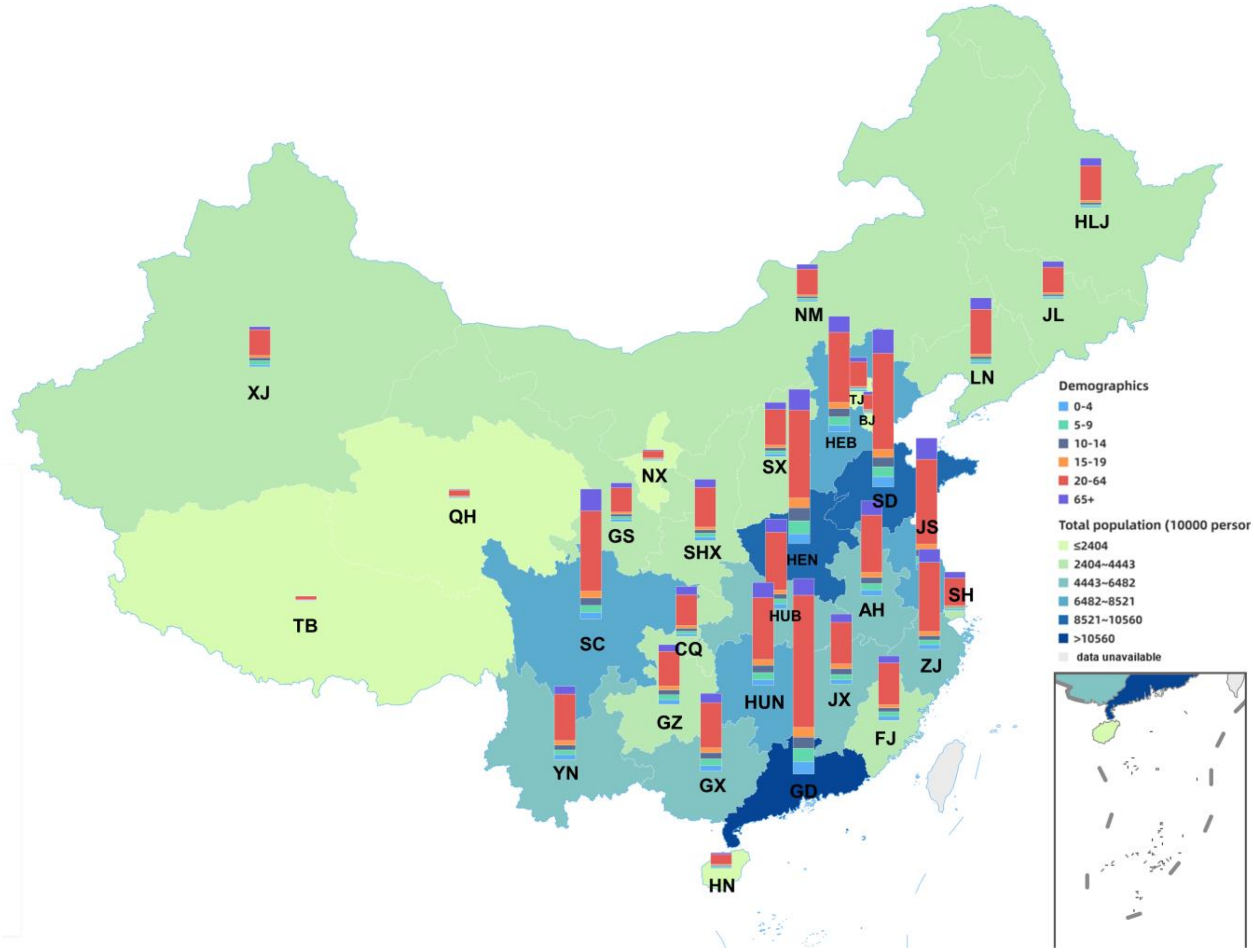

Figure **S2**. Population and demographics distribution in China. Abbreviation: BJ: Beijing, TJ: Tianjin, HEB: Hebei, SX: Shanxi, NM: Inner Mongolia, LN: Liaoning, JL: Jilin, HLJ: Heilongjiang, SH: Shanghai, JS: Jiangsu, ZJ: Zhejiang, AH: Anhui, FJ: Fujian, JX: Jiangxi, SD: Shandong, HEN: Henan, HUB: Hubei, HUN: Hunan, GD: Guangdong, GX: Guangxi, HN: Hainan, CQ: Chongqing, SC: Sichuan, GZ: Guizhou, YN: Yunnan, TB: Xizang, SHX: Shaanxi, GS: Gansu, QH: Qinghai, NX: Ningxia, XJ: Xinjiang.

Province	Energy Household (%)	Energy Eating-out (%)	Energy Food waste (%)	Energy Total Supply (10^{12} kcal)	Protein Household (%)	Protein Eating-out (%)	Protein Food waste (%)	Protein Total Supply (10^{4} ton)	Calcium Household (%)	Calcium Eating-out (%)	Calcium Food waste (%)	Calcium Total Supply (10^{3} ton)
BJ	80%	10%	11%	2.31E+01	76%	12%	12%	1.16E+02	84%	7%	9%	9.32E+00
TJ	82%	9%	8%	1.52E+01	79%	11%	10%	7.83E+01	84%	7%	8%	5.63E+00
HEB	86%	9%	5%	8.99E+01	82%	12%	6%	4.02E+02	85%	8%	6%	2.67E+01
SX	85%	9%	6%	3.85E+01	80%	13%	7%	1.65E+02	85%	9%	6%	1.17E+01
NM	85%	7%	7%	3.39E+01	82%	10%	8%	1.55E+02	85%	8%	7%	9.63E+00
LN	84%	9%	7%	4.91E+01	80%	11%	9%	2.43E+02	84%	8%	8%	1.62E+01
JL	86%	9%	5%	2.79E+01	82%	12%	7%	1.28E+02	84%	9%	7%	7.98E+00
HLJ	86%	9%	5%	3.69E+01	81%	12%	7%	1.70E+02	84%	9%	7%	1.06E+01
SH	79%	9%	12%	2.83E+01	77%	10%	13%	1.59E+02	82%	7%	11%	1.01E+01
JS	81%	9%	10%	9.73E+01	78%	10%	12%	5.13E+02	81%	8%	11%	3.09E+01
ZJ	82%	8%	10%	8.07E+01	79%	10%	11%	4.24E+02	81%	8%	11%	2.40E+01
AH	84%	8%	8%	7.94E+01	81%	10%	10%	3.95E+02	83%	8%	9%	2.28E+01
FJ	82%	9%	10%	4.87E+01	79%	10%	11%	2.63E+02	80%	9%	11%	1.40E+01
JX	84%	9%	7%	5.41E+01	81%	11%	8%	2.68E+02	83%	9%	8%	1.51E+01
SD	83%	9%	8%	1.13E+02	79%	12%	9%	5.53E+02	84%	8%	8%	3.75E+01
HEN	84%	9%	7%	1.12E+02	79%	12%	8%	5.04E+02	83%	9%	8%	3.27E+01
HUB	82%	10%	8%	6.24E+01	79%	11%	9%	3.18E+02	82%	9%	9%	1.88E+01
HUN	85%	8%	7%	8.61E+01	83%	10%	7%	4.20E+02	83%	9%	8%	2.25E+01
GD	83%	8%	8%	1.55E+02	82%	9%	9%	9.05E+02	82%	8%	10%	4.67E+01
GX	86%	9%	5%	5.74E+01	82%	12%	6%	2.70E+02	83%	11%	7%	1.43E+01
HN	83%	9%	7%	1.10E+01	82%	10%	8%	6.48E+01	82%	10%	9%	3.19E+00
CQ	83%	8%	9%	4.17E+01	81%	10%	10%	2.12E+02	82%	8%	10%	1.29E+01
SC	83%	8%	9%	1.10E+02	81%	10%	9%	5.45E+02	82%	8%	10%	3.18E+01
GZ	83%	11%	6%	3.63E+01	78%	14%	8%	1.72E+02	78%	13%	10%	9.27E+00
YN	85%	10%	5%	5.02E+01	81%	12%	7%	2.40E+02	80%	11%	9%	1.26E+01
TB	87%	7%	5%	5.17E+00	82%	11%	7%	2.08E+01	81%	12%	7%	9.11E-01
SHX	83%	10%	7%	4.27E+01	77%	13%	10%	1.87E+02	82%	10%	9%	1.25E+01
GS	86%	8%	5%	3.12E+01	81%	12%	7%	1.32E+02	84%	9%	6%	8.13E+00
QH	82%	11%	7%	5.44E+00	76%	15%	9%	2.45E+01	75%	16%	10%	1.14E+00
NX	83%	10%	7%	7.14E+00	77%	14%	9%	3.20E+01	80%	11%	9%	2.01E+00
XJ	84%	9%	7%	3.05E+01	79%	12%	9%	1.37E+02	83%	9%	8%	8.88E+00
Nationwide	84%	9%	8%	1.66E+03	80%	11%	9%	8.24E+03	83%	9%	9%	4.92E+02

Table **S6** (a). Nutrient supply in practical market (Energy, Protein, Calcium)

Province	Iron Household (%)	Iron Eating-out (%)	Iron Food waste (%)	Iron Total Supply (10^{3} ton)	Zinc Household (%)	Zinc Eating-out (%)	Zinc Food waste (%)	Zinc Total Supply (10^{3} ton)	Folate Household (%)	Folate Eating-out (%)	Folate Food waste (%)	Folate Total Supply (ton)
BJ	59%	8%	33%	9.12E+02	78%	12%	10%	1.15E+02	75%	8%	16%	8.73E+00
TJ	63%	8%	28%	5.72E+02	80%	12%	8%	7.51E+01	78%	8%	14%	5.56E+00
HEB	70%	9%	21%	2.81E+03	83%	12%	5%	4.08E+02	80%	9%	11%	2.72E+01
SX	65%	10%	26%	1.25E+03	81%	13%	6%	1.72E+02	78%	10%	12%	1.17E+01
NM	66%	7%	27%	1.10E+03	84%	10%	6%	1.60E+02	78%	8%	14%	9.58E+00
LN	65%	8%	27%	1.71E+03	81%	12%	7%	2.36E+02	78%	9%	14%	1.63E+01
JL	71%	10%	20%	8.61E+02	83%	12%	6%	1.29E+02	80%	9%	11%	8.37E+00
HLJ	70%	9%	21%	1.17E+03	83%	12%	5%	1.70E+02	80%	9%	11%	1.13E+01
SH	63%	8%	29%	1.08E+03	77%	11%	12%	1.44E+02	76%	8%	16%	1.01E+01
JS	63%	8%	29%	3.61E+03	78%	11%	10%	4.80E+02	76%	8%	15%	3.32E+01
ZJ	65%	8%	27%	2.79E+03	80%	11%	10%	3.92E+02	77%	8%	15%	2.51E+01
AH	69%	8%	23%	2.72E+03	82%	10%	8%	3.81E+02	80%	8%	13%	2.54E+01
FJ	67%	8%	25%	1.67E+03	79%	11%	10%	2.41E+02	77%	9%	14%	1.52E+01
JX	71%	9%	20%	1.72E+03	82%	11%	7%	2.60E+02	80%	9%	11%	1.63E+01
SD	62%	8%	30%	4.11E+03	80%	12%	8%	5.31E+02	77%	9%	15%	3.77E+01
HEN	64%	9%	27%	3.82E+03	81%	12%	7%	5.12E+02	77%	9%	13%	3.51E+01
HUB	65%	9%	26%	2.18E+03	80%	12%	8%	3.06E+02	77%	9%	14%	2.06E+01
HUN	72%	8%	20%	2.77E+03	83%	10%	6%	4.10E+02	81%	8%	11%	2.59E+01
GD	69%	7%	24%	6.23E+03	82%	10%	8%	8.19E+02	80%	7%	13%	5.73E+01
GX	71%	10%	19%	1.64E+03	83%	12%	5%	2.69E+02	79%	11%	10%	1.52E+01
HN	70%	8%	23%	4.44E+02	82%	11%	7%	5.74E+01	80%	8%	12%	4.08E+00
CQ	65%	7%	28%	1.50E+03	81%	10%	9%	2.10E+02	79%	7%	14%	1.42E+01
SC	67%	7%	26%	3.85E+03	82%	10%	8%	5.45E+02	79%	8%	13%	3.59E+01
GZ	64%	11%	25%	1.18E+03	80%	14%	7%	1.77E+02	75%	12%	13%	1.06E+01
YN	67%	10%	23%	1.61E+03	82%	12%	6%	2.46E+02	77%	11%	12%	1.46E+01
TB	73%	10%	18%	1.27E+02	85%	10%	5%	2.35E+01	76%	12%	12%	9.88E-01
SHX	60%	9%	31%	1.45E+03	79%	13%	8%	1.94E+02	75%	10%	15%	1.31E+01
GS	73%	10%	18%	8.81E+02	83%	12%	6%	1.39E+02	80%	10%	10%	8.31E+00
QH	60%	13%	27%	1.52E+02	79%	14%	7%	2.63E+01	62%	19%	19%	1.02E+00
NX	67%	10%	23%	2.48E+02	79%	13%	8%	3.42E+01	77%	10%	13%	2.39E+00
XJ	69%	9%	22%	9.69E+02	82%	11%	7%	1.50E+02	77%	10%	13%	8.74E+00
Nationwide	67%	8%	25%	5.75E+04	81%	11%	8%	8.04E+03	78%	9%	13%	5.34E+02

Table **S6** (b). Nutrient supply in practical market (Iron, Zinc, Folate)

Province	Vitamin b12 Household (%)	Vitamin b12 Eating-out (%)	Vitamin b12 Food waste (%)	Vitamin b12 Total Supply (ton)	Vitamin a Household (%)	Vitamin a Eating-out (%)	Vitamin a Food waste (%)	Vitamin a Total Supply (ton)	Vitamin c Household (%)	Vitamin c Eating-out (%)	Vitamin c Food waste (%)	Vitamin c Total Supply (ton)
BJ	68%	16%	15%	5.64E+01	75%	11%	14%	1.31E+01	86%	5%	9%	5.61E+03
TJ	71%	16%	13%	3.67E+01	77%	11%	12%	8.08E+00	87%	5%	8%	3.61E+03
HEB	69%	21%	10%	1.48E+02	78%	13%	9%	3.66E+01	87%	6%	7%	1.64E+04
SX	59%	29%	12%	4.99E+01	75%	16%	9%	1.44E+01	86%	7%	7%	6.91E+03
NM	71%	18%	11%	5.68E+01	77%	13%	10%	1.27E+01	86%	6%	8%	5.20E+03
LN	70%	17%	13%	1.04E+02	76%	12%	12%	2.30E+01	86%	6%	8%	9.69E+03
JL	69%	20%	11%	5.01E+01	77%	14%	10%	1.16E+01	86%	7%	7%	4.90E+03
HLJ	70%	20%	10%	6.74E+01	78%	13%	9%	1.57E+01	86%	6%	7%	6.65E+03
SH	75%	10%	15%	1.02E+02	77%	9%	14%	1.85E+01	84%	6%	10%	5.40E+03
JS	74%	12%	14%	3.00E+02	79%	10%	12%	5.86E+01	84%	7%	9%	1.64E+04
ZJ	75%	11%	14%	2.39E+02	78%	10%	12%	4.33E+01	84%	7%	9%	1.29E+04
AH	77%	12%	12%	2.23E+02	81%	9%	10%	4.50E+01	85%	6%	8%	1.32E+04
FJ	76%	11%	13%	1.57E+02	78%	10%	12%	2.79E+01	83%	8%	10%	7.33E+03
JX	75%	14%	11%	1.36E+02	79%	11%	10%	2.73E+01	85%	7%	8%	8.31E+03
SD	71%	17%	12%	2.48E+02	77%	12%	11%	5.44E+01	86%	6%	8%	2.25E+04
HEN	68%	20%	12%	2.07E+02	77%	13%	10%	5.05E+01	85%	7%	8%	1.94E+04
HUB	72%	16%	12%	1.47E+02	77%	12%	11%	3.11E+01	84%	7%	9%	1.11E+04
HUN	79%	13%	9%	2.21E+02	82%	10%	8%	4.48E+01	86%	7%	8%	1.36E+04
GD	84%	8%	9%	7.01E+02	85%	7%	9%	1.28E+02	84%	6%	9%	2.60E+04
GX	64%	23%	12%	9.11E+01	71%	17%	12%	1.93E+01	85%	8%	7%	8.17E+03
HN	85%	8%	7%	5.27E+01	85%	7%	7%	9.40E+00	83%	8%	9%	1.70E+03
CQ	75%	13%	12%	1.03E+02	80%	9%	10%	2.33E+01	85%	6%	9%	7.33E+03
SC	76%	13%	11%	2.73E+02	81%	9%	10%	6.07E+01	85%	6%	9%	1.81E+04
GZ	65%	24%	11%	6.65E+01	74%	17%	10%	1.54E+01	82%	9%	9%	5.59E+03
YN	70%	19%	10%	1.03E+02	77%	14%	10%	2.27E+01	83%	9%	9%	7.27E+03
TB	65%	26%	9%	5.92E+00	63%	25%	12%	9.52E-01	74%	15%	11%	3.22E+02
SHX	55%	26%	18%	6.32E+01	71%	16%	13%	1.67E+01	84%	7%	9%	7.28E+03
GS	67%	24%	9%	4.40E+01	77%	15%	8%	1.09E+01	85%	7%	8%	4.69E+03
QH	65%	25%	10%	1.01E+01	55%	30%	15%	1.29E+00	61%	21%	18%	3.79E+02
NX	69%	21%	10%	1.47E+01	77%	13%	10%	3.57E+00	85%	6%	9%	1.58E+03
XJ	68%	21%	11%	5.12E+01	75%	15%	11%	1.17E+01	84%	7%	9%	4.95E+03
Nationwide	75%	14%	11%	4.22E+03	79%	11%	10%	8.80E+02	85%	7%	8%	2.83E+05

Table **S6** (c). Nutrient supply in practical market (Vitamins)

Province	this study (2019)	Yin et al., 2022 (average: 2015-2020)	this study (2019)	Yin et al., 2022 (average: 2015-2020)
	Energy	Energy	Protein	Protein
TJ	0.56	0.36	0.10	0.36
HEB	2.06	1.40	0.61	1.48
JS	2.01	1.72	1.78	1.94
SD	2.39	1.78	2.58	1.89
GX	1.56	1.50	1.54	1.03
LN	1.81	0.88	2.03	0.92
HN	1.02	0.93	1.68	1.14
ZJ	0.59	0.44	0.89	0.62
FJ	0.66	0.31	1.55	0.40
GD	0.45	0.36	0.68	0.30
SH	0.23	0.19	0.23	0.14

Table **S7.** Local energy and protein supply adequacies of 11 coastal provinces in China.

References:

CAY, 2020. China Agricultural Yearbook (2020). China agricultural yearbook editorial committee.

CFIY, 2020. China food industry yearbook (2020). Federation, C.F.I. (ed).

Chapagain, A.K., Hoekstra, A.Y., 2003. Virtual water flows between nations in relation to trade in livestock and livestock products. UNESCO-IHE Value of water research report series. https://www.waterfootprint.org/media/downloads/Report13.pdf

FAOSTAT, 2020. FAOSTAT statistics database. Nations, F. A.O. (ed). https://www.fao.org/faostat/en/.

Gini, C., 1921. Measurement of Inequality of Incomes. The Economic Journal 31, 124–126.

Li, C., Bremer, P., Harder, M.K., Lee, M.SW., Parker, K., Gaugler, E.C., Mirosa, M., 2022. A systematic review of food loss and waste in China: Quantity, impacts and mediators. Journal of Environmental Management 303, 114092.

Lu, S., Cheng, G., Li, T., Xue, L., Liu, X., Huang, J., Liu, G., 2022. Quantifying supply chain food loss in China with primary data: A large-scale, field-survey based analysis for staple food, vegetables, and fruits. Resour. Conserv. Recycl 177, 106006.

NBSC, 2021. Seventh National Census Communiqué. National Bureau of Statistics of China (ed).

NBSC, 2020. Annual statistics data by provinces. National Bureau of Statistics of China (ed).

Niu, Z., Ng, S.J., Li, B., Han, J., Wu, X., Huang, Y., 2022. Food waste and its embedded resources loss: A provincial level analysis of China. Science of The Total Environment 823, 153665.

Nutrient Data Laboratory, 2016. USDA national nutrient database for standard reference, release 28 (slightly revised). USDA http://www.ars.usda.gov/nea/ bhnrc/mafcl.

Schreiber, K., Hickey, G.M., Metson, G.S., Robinson, B.E., MacDonald, G.K., 2021. Quantifying the foodshed: a systematic review of urban food flow and local food self-sufficiency research. Environ. Res. Lett. 16, 023003.

Wang, L., Gao, B., Hu, Y., Huang, W., Cui, S., 2020. Environmental effects of sustainability-oriented diet transition in China. Resour. Conserv. Recycl 158, 104802.

Wang, R., Liu, G., Zhou, L., Yang, Z., Tang, Z., Lu, S., Zhao, M., Sun, H., Ma, C., Cheng, G., 2023. Quantifying food loss along the animal products supply chain in China with large-scale field-survey based primary data. Resour. Conserv. Recycl 188, 106685.

Wood, S.A., Smith, M.R., Fanzo, J., Remans, R., DeFries, R.S., 2018. Trade and the equitability of global food nutrient distribution. Nat Sustain 1, 34–37.

Xin, L., Wang, L., Liu, A., 2018. Characteristics of regional production and consumption balance of feed grains in China and policy implications. Journal of Natural Resources 33, 965–977.

Yang, L., 2009. Analysis of China’s grain supply and demand balance. Shandong University of Technology, MA Thesis.

Zeng, Q., Zeng, Y., 2018. Eating out and getting fat? A comparative study between urban and rural China. Appetite 120, 409–415.

Yin, W.; Yu, H.; Wang, Y.; Qiu, R.; Han, L., 2022. Spatial Differences of Nutrient Adequacy in Coastal Areas of China. Nutrients, 14 (22), 4763. https://doi.org/10.3390/nu14224763.
